# Effects of Ambient Temperature and State of Galvanized Layer on Corrosion of Galvanized Steel in High-Humidity Neutral Atmosphere

**DOI:** 10.3390/ma16103656

**Published:** 2023-05-11

**Authors:** Yusong Liang, Bin He, Guo Fu, Shoujun Wu, Bin Fan

**Affiliations:** 1 College of Water Resources and Architectural Engineering, Northwest A&F University, Yangling, Xianyang 712100, China; lys18707909156@163.com (Y.L.);; 2 Key Laboratory of Agricultural Soil and Water Engineering in Arid and Semiarid Areas, Ministry of Education, Northwest A&F University, Yangling, Xianyang 712100, China

**Keywords:** galvanized steel, neutral atmospheric environment, temperature, atmospheric corrosion, mechanical properties, chemical products

## Abstract

Galvanized steel is a cost-effective and corrosion-resistant material with high strength, making it a popular choice for various engineering applications. In order to investigate the effects of ambient temperature and galvanized layer state on the corrosion of galvanized steel in a high-humidity neutral atmosphere environment, we placed three types of specimens (Q235 steel, undamaged galvanized steel, damaged galvanized steel) in a neutral atmosphere environment with a humidity of 95% at three different temperatures (50 °C, 70 °C, and 90 °C) for testing. The corrosion behavior of specimens under simulated high-temperature and high-humidity conditions was studied using weight changes, macroscopic and microscopic observations, and analysis of the corrosion products of the specimens before and after corrosion. Emphasis was placed on examining the effects of temperature and damage to the galvanized layer on the corrosion rate of the specimens. The findings indicated that damaged galvanized steel retains good corrosion resistance at 50 °C. However, at 70 °C and 90 °C, the damage to the galvanized layer will accelerate the corrosion of the base metal.

## 1. Introduction

Zinc is frequently employed as a protective layer for carbon steel components in environments similar to the atmosphere, water, and soil to prevent corrosion. This is achieved by utilizing sacrificial anodes and the cathodic protection principle to safeguard the base metal from damage. As a result, there is considerable global demand for zinc consumption and production. With science and technology continuing to advance, research is increasingly focusing on the corrosion of galvanized steel. Foreign scholars began studying the atmospheric corrosion of galvanized steel earlier than their peers in other parts of the world. In 1968, Guttman conducted a study on the impact of various environments on galvanized steel [1]. In 1993, Odnevall et al. focused on the corrosion of galvanized steel in a marine atmosphere. They found that the corrosion products formed due to the formation of water films on the surface consisted of NaZn_4_Cl(OH)_6_SO_4_·6H_2_O [2]. Persson et al. compared the corrosion products of hot-dip galvanized steels from Europe, East Asia, and North America under natural exposure, ranking their sulfate corrosion product content in order of magnitude [3]. Del Angel studied the atmospheric corrosion behavior of galvanized steel in three cities in Chile and Mexico under natural exposure, showing that the distribution of corrosion was not uniform and sulfur dioxide accelerated the corrosion rate of galvanized steel [4]. Syed S. monitored the dynamic data of galvanized steel under natural exposure in Saudi Arabia for four consecutive years and found that the corrosion rate function was highly correlated with chloride, sulfate, and humidity [5]. Saricimen H analyzed galvanized steel under natural corrosion for 15 months in three types of environments—atmospheric, soil, and splash—along the Arabian Gulf coast, showing that the splash zone is significantly more corrosive than other areas [6].

In China, Zheng et al. studied the corrosion of carbon steel and galvanized steel in natural exposure for three years under different humidity and temperature environments in the southwest [7]. Gu’s research on the natural corrosion rate of galvanized steel in Northeast China found that snow accumulation and the formation of dense corrosion products can inhibit the occurrence of corrosion [8]. Zhang et al. conducted an initial study on the corrosion of galvanized steel in the environment of Qinghai Lake, focusing on the effect of Cl^-^ on the composition of corrosion products [9].

Although natural exposure experiments bring the most realistic and reliable data, there are disadvantages, such as the long cycle time. Experimental results are greatly affected by the factors of the experimental site, especially in the less corrosive atmospheric environment, where the complete corrosion cycle of galvanized steel is more time-consuming. Therefore, an increasing number of researchers have been using indoor simulation acceleration experiments and prediction models to study the atmospheric corrosion changes of galvanized steel. In 1995, Johansson et al. carried out an accelerated experimental design on carbon steel and galvanized steel and compared it with the natural exposure environment [10]. Qiao et al. simulated a coastal industrial atmospheric environment through wet and dry cycle tests and investigated the influence of corrosion product structure on corrosion kinetics [11]. Wang et al. simulated the atmospheric environment in Guangzhou, China, by preparing chemical solutions and obtaining the corrosion law of galvanized steel in alternating wet and dry conditions [12]. Regarding the establishment of prediction models, in 1992, Spence et al. estimated the corrosion rate of galvanized steel by describing the growth and dissolution rates of the basic zinc carbonate film [13]. Two years later, they proposed the use of models such as RAMD, EM, and MM-4 to predict the atmospheric corrosion of galvanized steel [14]. Dubuisson E studied localized corrosion of galvanized steel using chemical solutions and developed an empirical model to predict the corrosion rate based on variables such as corrosion time, temperature, electrolytes, chlorides, and sulfates [15]. Some researchers found that long-term atmospheric corrosion data for zinc conforms to the general equation C = At^n^ [16,17,18].

Current research on galvanized steel’s atmospheric corrosion primarily concentrates on natural atmospheric conditions. Marine and industrial atmospheric environments are the primary research focus in this regard. However, research on the atmospheric corrosion testing of galvanized steel in neutral atmospheric environments such as rural and urban atmospheres is inadequate. Notably, there are deficiencies in the accelerated corrosion testing of galvanized steel in such environments. Our study aimed to investigate the corrosion behavior of galvanized steel in a simulated neutral atmosphere with high humidity. The research focused on the effects of temperature and galvanized layer damage on the corrosion rate of the specimen. The results can provide data support and assistance for future indoor simulation acceleration experiments of undamaged or damaged galvanized steel in a neutral atmospheric environment.

## 2. Materials and Methods

### 2.1. Experimental Materials

**Figure 1:**
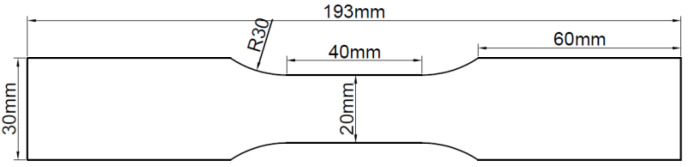
Schematic diagram of experimental specimens.

**Figure 2:**
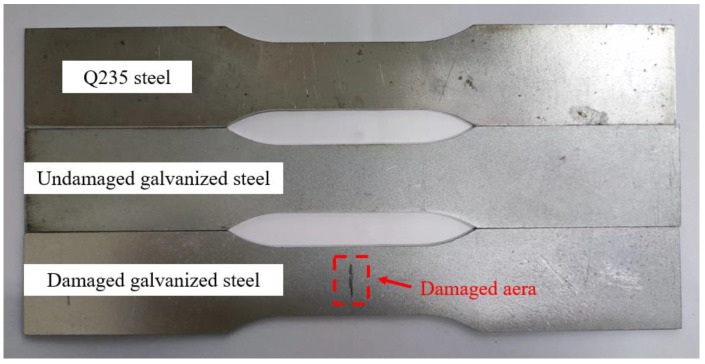
Schematic diagram of specimens.

The specimens utilized in the test were fabricated from Q235 steel plate and hot-dip galvanized steel plate using a CNC wire cutting machine. The size of each test piece was designed according to ISO 6892-1 [19], as illustrated in Figure 1. Prior to use, the specimens were degreased with acetone and kept in an oven. The chemical composition of the steel plate specimens was analyzed by a Spectro spark direct reading spectrometer (SPECTRO MAX, SPECTRO Analytical Instruments GmbH, Kleve, Germany) to determine the mass fraction of the main alloy elements, which for the Q235 steel had the following chemical composition (mass fraction, %): C 0.18, Si 0.25, Mn 0.5, P 0.018, S 0.016, Fe balance. The galvanized steel specimens were manufactured by hot-dip plating in accordance with ISO 1460 [20]. Based on the manufacturer’s data, the zinc layer thickness of galvanized steel was 50 μm, which adhered to ISO 1461 [21]. The thickness of the Q235 steel specimen was 0.95 ± 0.01 mm, and that of the galvanized steel specimen was 1.16 ± 0.01 mm. The experimental specimens were categorized into three groups: (1) Q235 carbon steel, (2) undamaged galvanized steel, and (3) damaged galvanized steel. The damaged galvanized steel was produced using the undamaged galvanized steel as the prototype. To create a 10 mm-long gap in the center of one side of the undamaged galvanized steel and destroy the zinc layer, a surface scratch test needle was used to make a damaged notch with a width of approximately 1 mm and a depth of approximately 0.1 mm. The damaged area was less than 0.2% of the total area of the galvanized steel specimen, rendering it negligible. Therefore, the specific specification requirements for scratches on damaged galvanized steel specimens do not have to be too precise. The actual images of the three specimens are displayed in Figure 2.

### 2.2. Corrosion Test

**Figure 3:**
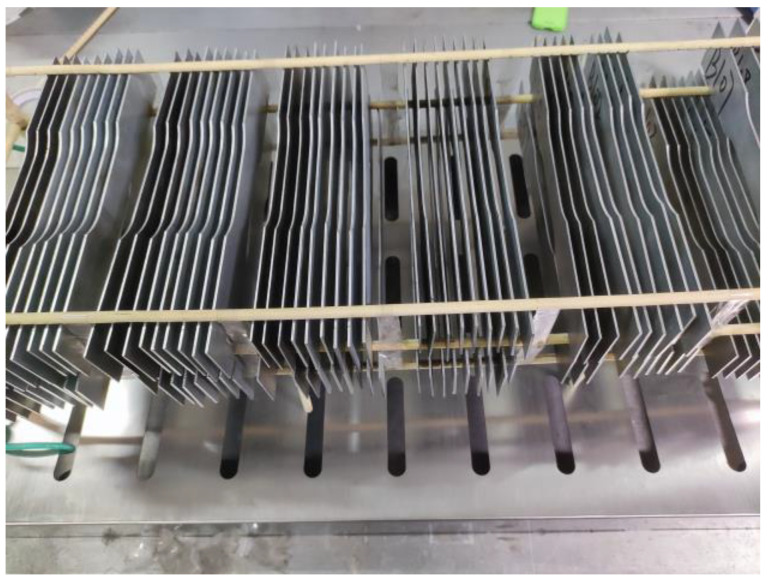
Schematic diagram of specimen placement.

Some studies have shown that the corrosion rate of galvanized steel in solution increases significantly when the temperature exceeds 60 °C [22,23]. Compared with the marine atmospheric environment and industrial atmospheric environment, since this study simulates a rural atmospheric environment, the concentration of Cl^−^ and SO_2_ in the environment is lower, so the corrosion rate of the specimens is slower. In order to accelerate the corrosion process of the specimens, the temperatures of 50 °C, 70 °C, and 90 °C were simulated in a damp heat test chamber (KZ-TH-80B, Dongguan Ke Zheng Instrument Co., Ltd., Dongguan, China), distilled water was used as the test solution, and the relative humidity was 95%. Legault and Pearson found that the sky-facing surface of galvanized steel specimens corroded faster than the ground-facing surface [24]. Therefore, to avoid the galvanized steel with a broken notch on only one side being affected by the above reasons, all specimens were placed on custom-made supports perpendicular to the horizontal plane, as shown in Figure 3. Before the experiment, epoxy resin was used to protect the cutting edges of the specimens to prevent edge corrosion. The experiment involved taking out a batch of specimens every 15 days, with a continuous testing period of 5 months.

### 2.3. Mass Loss Analysis

The rust removal solution was prepared according to ISO 8407 [25]. For Q235 steel, 500 mL of hydrochloric acid with ρ = 1.19 g/mL, 3.5 g of hexamethylenetetramine, and the remaining amount of distilled water were used to prepare a 1000 mL of solution. For galvanized steel, select 205 g of ammonium acetate and distilled water to prepare 1000 mL solution, followed by ultrasonic cleaning (KS-500DV, Shanghai Yitian Scientific Instrument Co., Ltd., Shanghai, China) at 20–25 °C environment for 15 min and the use of a brush to gently brush the surface corrosion products. The specimen was rinsed with distilled water after rust removal, rinsed with the use of alcohol, and finally dried in a drying oven for 24 h. The dried specimens were weighed using a high-precision balance (ME55/02, Mettler Toledo, Greifensee, Switzerland) with a mass accuracy of 0.1 mg. Three parallel specimens were used to average the weight loss values for each batch.

The weight loss method was used to express the corrosion rate of the specimens. The average corrosion rate *V*_m_ of specimens expressed in g·cm^−2^·d^−1^ is calculated according to Formula (1):

(1) $$V_{m}=\frac{m_{0}-m_{i}}{St_{i}}$$

In the formula, m_0_ is the initial specimen mass, g; *m_i_* is the specimen mass on the *i*-th day, g; *S* is the total surface area of the specimen, cm^2^; *t_i_* is the corrosion time of the specimen on the *i*-th day, d, where *t*_0_ = 0, *t*_15_ = 15, *t*_30_ = 30, *t*_45_ = 45, etc., *i* = 0, 15, 30, 45… [9].

### 2.4. Analysis of Specimen Surface Morphology and Corrosion Products

**Figure 4:**
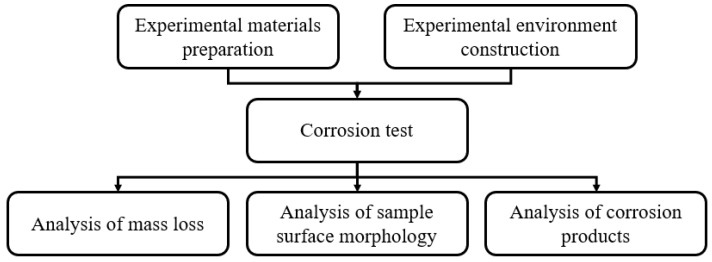
Organization chart.

Take each specimen in the test chamber to photograph the macroscopic corrosion morphology of the specimen, and then take the center of the specimen cut into 5 mm × 5 mm specimens, using a scanning electron microscope (TESCAN VEGA 3 XMU, TESCAN ORSAY HOLDING, a.s, Brno, Czech Republic) to observe the microscopic morphology of corrosion products on the surface of the specimen. An X-ray diffractometer (D8 ADVANCE A25, Bruker, Billerica, MA, USA) was used to identify the corrosion product composition (Cu target with a voltage of 40 kV and a current of 40 mA), and diffraction angle 2θ was scanned from 10° to 80° at a scan rate of 0.15°/s. All experimental steps are shown in Figure 4.

## 3. Results and Discussion

### 3.1. Atmospheric Corrosion Kinetics of Galvanized Steel

**Figure 5:**
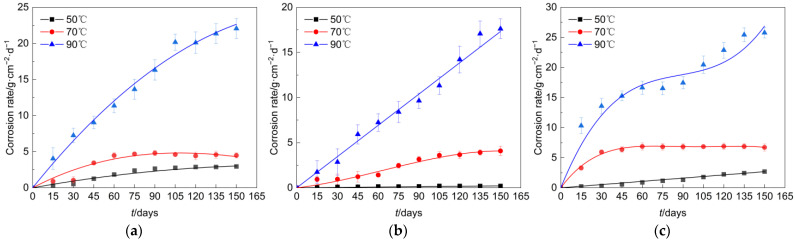
Corrosion rate of each specimen. (**a**) Q235 steel. (**b**) Undamaged galvanized steel. (**c**) Damaged galvanized steel.

In the simulated environment of this study, the corrosion rate curves of Q235 steel, undamaged galvanized steel, and damaged galvanized steel with time were shown in Figure 5. Overall, the corrosion rate of all types of specimens was basically in the rising stage, but the maximum corrosion rate of different materials and the time point to reach the maximum corrosion rate had a large difference. At an elevated temperature of 50°C, the corrosion rate of the galvanized steel specimen that had not sustained any damage was comparatively lower, whereas the corrosion rate of the damaged galvanized steel specimen was slightly higher when compared to that of the Q235 steel specimen. However, with an increase in temperature, the corrosion rate of the damaged galvanized steel specimen surpassed that of the Q235 steel specimen. We believed that this phenomenon may be due to the lower temperature case; the galvanized steel damage at the contact area of air was not large, and there was only a small amount of zinc and iron involved in the reaction, so the corrosion rate was lower in the early stage. As the temperature increased, the iron at the damage was constantly involved in the reaction, superimposed on the redox reaction in which zinc acts as an anode, so the corrosion rate increased as the test proceeded.

The increase in temperature often causes the intensification of corrosion on the specimen and produces more oxidation products faster. The dense oxidation products will slow down the further corrosion of the specimens, similar to the behavior of these specimens at 70 °C [16,17]. However, compared to the conditions of 50 °C and 70 °C, the corrosion rate of all specimens in the 90 °C case increased significantly and did not show a slowing trend. We believed this was due to the fact that the corrosion products generated at 90 °C in the specimens did not effectively protect the base metal. Although the corrosion rate of the galvanized steel material was slightly stable from day 60 to day 90 of the test, the zinc layer did not provide durable protection, allowing the base metal to be heavily involved in the corrosion reaction process.

### 3.2. Chemical Analysis of Corrosion Products

**Figure 6:**
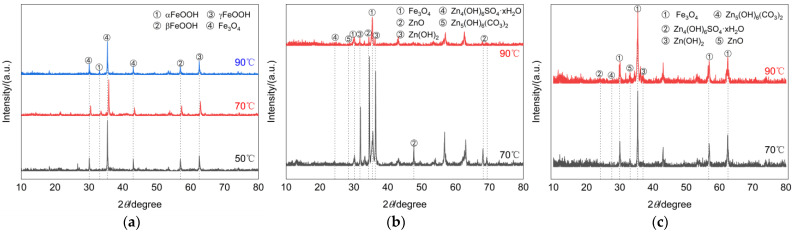
XRD patterns of corrosion products. (**a**) Q235 steel. (**b**) Undamaged galvanized steel. (**c**) Damaged galvanized steel.

- Generally speaking, the atmospheric corrosion behavior of carbon steel-like metal materials is often closely related to temperature, humidity, and the atmospheric environment. At the beginning of corrosion, the surface of the galvanized layer often forms a water film due to humidity, which leads to the formation of Zn(OH)_2_ on the surface, causing a decrease in PH, which combines with CO_2_, SO_2_, and other gases present in the air to form a new oxide. For example, combine with CO_2_ to form Zn_5_(OH)_6_(CO_3_)_2_, etc., and combine with SO_2_ to form Zn_4_(OH)_6_SO_4_. The oxide formed in turn reduces the contact between the base metal and the corrosive environment, thus inhibiting the occurrence of corrosion. However, in the simulated environment of this study, on the one hand, the ambient humidity was high and the surface of the galvanized layer was prone to form a liquid film; on the other hand, the ambient temperature was high and SO_2_, CO_2_, and Cl^-^ were present in the air.
- The specimens of each type on the 60th day of the test were selected for chemical analysis of corrosion products. Since the corrosion products of damaged and undamaged galvanized steel were extremely small at 50 °C, no specimens were taken for these two types of specimens. Figure 6 shows the XRD physical phase analysis of the surface corrosion products of the three types of specimens after corrosion at different temperatures. It can be seen that the composition of the surface corrosion products of galvanized steel was slightly different at different temperatures, especially for galvanized steel as a material. At 70 °C, the peak intensities of ZnO and Zn(OH)_2_ in the corrosion products of undamaged galvanized steel are relatively high, and there are also lower peak intensities of Zn_5_(OH)_6_(CO_3_)_2_ and Zn_4_(OH)_6_SO_4_ xH_2_O. However, at 90 °C, the peak intensity of ZnO in the corrosion products drops sharply, while that of Fe_3_O_4_ increases significantly. Based on this phenomenon, we conclude that at 90 °C, the base metal of galvanized steel is already undergoing corrosion. For damaged galvanized steel, there are oxidation products of both iron and zinc due to the destruction of the galvanized layer. Its corrosion products were mainly Fe_3_O_4_, ZnO, and Zn(OH)_2_, accompanied by Zn_4_(OH)_6_SO_4_ xH_2_O and Zn_5_(OH)_6_(CO_3_)_2_. As the temperature increased, the intensity of the peaks corresponding to the corrosion products ZnO and Zn(OH)_2_ gradually became more and more obvious, indicating that the increase in temperature also caused the zinc in other undamaged regions to participate more actively in the oxidation reaction. The corrosion products of Q235 steel were mainly Fe_3_O_4_, αFeOOH, βFeOOH, and γFeOOH, and their peak intensity increased to varying degrees with the increase in temperature.

### 3.3. Analysis of Rust Layer Morphology

#### 3.3.1. Macroscopic Morphology

**Figure 7:**
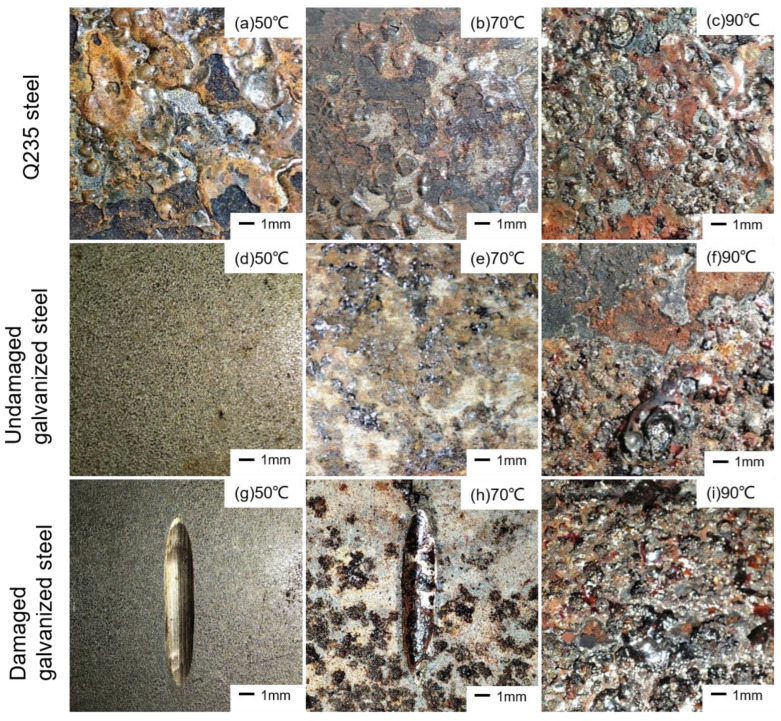
Macroscopic morphology of specimens.

Figure 7 shows the macroscopic morphology of the three types of specimens at different temperature simulation environments on the 60th day of the test. It could be obtained that at 50 °C, large yellow corrosion areas had appeared on the surface of the Q235 steel specimen. With the increase in temperature, the corrosion products turned black and bubble-like. No obvious corrosion products were found in the undamaged galvanized steel specimens at 50 °C. As the temperature increased to 70 °C, some white powdery corrosion products with yellow-brown pitting also appeared on the surface of the specimens. At 90 °C, large bubble-like black corrosion products appeared on the surface of the undamaged galvanized steel, and some of the corrosion products came off in layers, exposing the yellow carbon steel base metal below. Damaged galvanized steel at 50 °C also did not observe obvious corrosion products. At 70 °C, brown spots of corrosion products appeared at the damage and they also appeared in other areas. When the temperature was further increased to 90 °C, a large number of black bubble-like corrosion products accumulated in the specimen damage, completely covering the damaged part, and at the same time, some black corrosion products also fell off in other areas. In general, as the temperature increased, more severe corrosion was observed on the specimens, the corrosion products were looser, and the corrosion product layer was more easily destroyed.

#### 3.3.2. Microscopic Morphology

**Figure 8:**
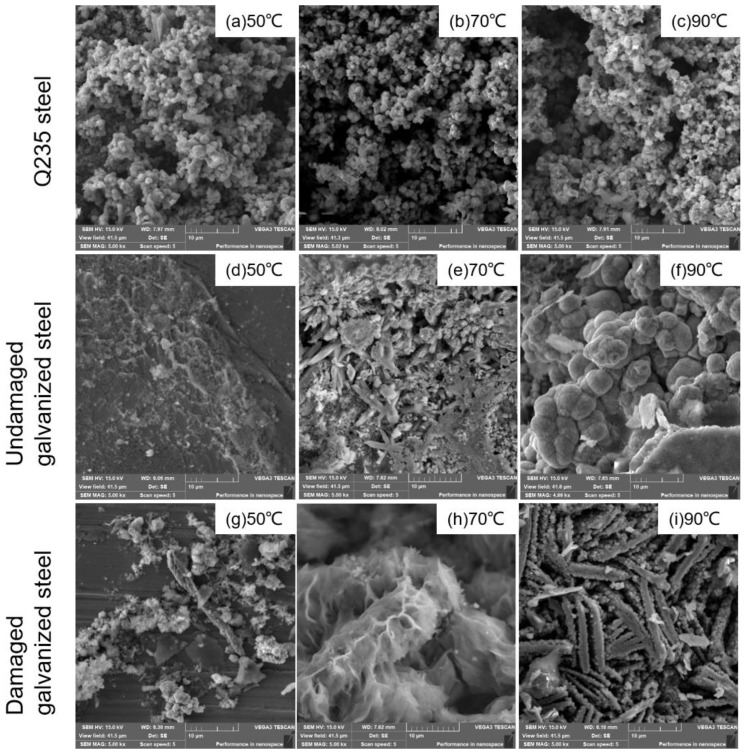
SEM images of corrosion products.

We included images of the surface corrosion products of the three types of specimens on the 60th day of the experiment in Figure 8. These images show the microscopic details of the corroded surface, helping to analyze the overall corrosion behavior. Figure 8a–c illustrated that the corrosion products of Q235 carbon steel exhibited a granular morphology under all temperature gradients. As the temperature increased, the inter-particle porosity also increased, indicating a less stable interconnection between the granular corrosion products. Based on Figure 8d–f, it was evident that under the 50 °C condition, the undamaged galvanized steel surface exhibited minimal corrosion product formation. However, at 70 °C, the surface of the component was gradually eroded, and cracks emerged. As the temperature increased to 90 °C, the undamaged galvanized steel corrosion products exhibited a spherical morphology. According to Figure 8g–i, at 50 °C, only a minimal amount of debris-like corrosion products was observed at the damaged site of galvanized steel. As the temperature increased to 70 °C, the rod-shaped corrosion products densely accumulated together. When the temperature rose to 90 °C, the corrosion products appeared as porous, strip-like structures with numerous gaps. The results indicated that the increase in temperature caused the corrosion products to accumulate and crack in the vertical outward direction at the corrosion interface of the specimens, which made the corrosion products more prone to detachment, leading to an overall increase in the corrosion rate of the specimens.

### 3.4. Discussion

Our findings on the corrosion of galvanized steel in a neutral atmospheric environment are in agreement with and supported by the research of other scholars. For example, in the course of investigating the corrosion of galvanized steel in a simulated environment, we observed the appearance of pitting corrosion on both undamaged and damaged galvanized steel surfaces at a temperature of 70 °C [26]. Additionally, XRD analysis revealed that the primary corrosion product of galvanized steel was ZnO, consistent with the observations of other researchers who subjected galvanized steel to long-term (over ten years) corrosion testing in rural atmospheric conditions [27,28,29,30]. In our study on galvanized steel corrosion, we observed significant cracking of the corrosion product layer in SEM images of the specimens. This observation is consistent with the surface morphology previously reported by Almeida on galvanized steel specimens exposed to a more aggressive urban atmosphere for 16 years [31]. Based on all test results, we summarize the performance of various specimens at different temperatures, as shown in Table 1. The contents of the table are as follows: “The relative corrosion rate based on the corrosion rate of Q235 steel specimen at 50 °C; (main corrosion product); the morphology of the corrosion product layer of the specimen under macroscopic and microscopic conditions”.

## 4. Conclusions

(1) In a neutral atmospheric environment with high humidity at 50 °C, the zinc layer of damaged galvanized steel can still effectively slow down the corrosion rate of the base metal. However, when the temperature rises to 70 °C and 90 °C, the damage to the galvanized layer will accelerate the corrosion of the base metal.
(2) In a neutral atmosphere with high humidity, the corrosion rate of galvanized steel will be significantly increased from 50 °C to 70 °C without changing the morphology or composition of its corrosion products. On the contrary, the effect on the corrosion rate of galvanized steel from 70 °C to 90 °C is not obvious, but the morphology and composition of its corrosion products change greatly.

This study provides valuable insights for indoor simulation acceleration experiments of undamaged or damaged galvanized steel in a neutral atmospheric environment, and future research can explore these details in further depth. Furthermore, this study was subject to experimental constraints that did not allow for a fine temperature gradient during testing, as well as a lack of electrochemical analysis, microscopic cross-sectional imaging, and EDS data analysis of the specimen surfaces. Next, we will further conduct more scientific and rigorous research on the mechanical properties of galvanized steel under the influence of different temperatures and the integrity of the zinc layer.

## Figures and Tables

**Table 1:** Comparison of test results of specimens.

	50 °C	70 °C	90 °C
Q235 steel	1; (Fe_3_O_4_); Loose	1.83; (Fe_3_O_4_); Loose	7.09; (Fe_3_O_4_); Loose
Undamaged galvanized steel	0.07; (×); ×	1.24; (ZnO); Dense	4.69; (Fe_3_O_4_, ZnO); Loose.
Damaged galvanized steel	0.66; (×); ×	3.10; (Fe_3_O_4_, ZnO); Dense	8.90; (Fe_3_O_4_, ZnO); Loose

## Data Availability

Not applicable.
